# Effects of Different Drying Methods on the Quality of Forest Ginseng Revealed Based on Metabolomics and Enzyme Activity

**DOI:** 10.3390/foods14152753

**Published:** 2025-08-07

**Authors:** Junjia Xing, Xue Li, Wenyu Dang, Limin Yang, Lianxue Zhang, Wei Li, Yan Zhao, Jiahong Han, Enbo Cai

**Affiliations:** College of Chinese Medicinal Material, Jilin Agricultural University, 2888 Xincheng Street, Changchun 130118, China; hill_0307@163.com (J.X.);

**Keywords:** forest ginseng, dry method, enzyme activity, widely targeted metabolomics, quality

## Abstract

Forest ginseng (FG) is a rare medicinal and culinary plant in China, and its drying quality is heavily dependent on the drying method. This study investigated the effects of traditional hot air drying (HAD) and the self-developed negative-pressure circulating airflow-assisted desiccator drying (PCAD) method on the quality of FG using metabolomics and enzyme activity. The results revealed that the enzyme activities of dried FG were reduced considerably. PCAD preserved higher enzyme activity than HAD. Metabolomics data demonstrate that HAD promotes the formation of primary metabolites (amino acids, lipids, nucleotides, etc.), whereas PCAD promotes the formation of secondary metabolites (terpenoids, phenolic acids, etc.). A change-transformation network was built by combining the metabolites listed above and their biosynthetic pathways, and it was discovered that these biosynthetic pathways were primarily associated with the mevalonate (MVA) pathway, lipid metabolism, phenylpropane biosynthesis, and nucleotide metabolism. It is also believed that these findings are related to the chemical stimulation induced by thermal degradation and the ongoing catalysis of enzyme responses to drought stress. The facts presented above will give a scientific basis for the selection of FG drying processes, as well as helpful references for increasing the nutritional quality of processed FG.

## 1. Introduction

Forest ginseng (FG), a rare and valuable plant native to China, possesses both medicinal and edible value. In 2023, it was approved as a food ingredient in China, further expanding its application scope. FG is rich in active components such as ginsenosides and ginseng polysaccharides [1,2], which confer significant pharmacological effects, including immune system enhancement, regulation of central nervous system function and anti-tumour properties, demonstrating immense potential in promoting human health [3,4]. In cultivation, FG seeds are sown in mountain forests and allowed to grow naturally in a wild state for 10–15 years [5]. No water or fertiliser intervention is required throughout the process, as it grows naturally by simulating the original forest environment, thereby maximising the retention of its natural characteristics. Due to its growth pattern and pharmacological effects being similar to those of wild mountain ginseng, it serves as an ideal alternative to wild mountain ginseng. Additionally, it can be cultivated on a large scale to provide a more abundant supply, meeting the market demand for high-quality ginseng products.

Among the several variables that affect product dried quality, the drying method is most crucial [6]. Therefore, the selection of suitable drying methods to meet the needs of consumers who value quality and nutrition is of paramount importance. At present, hot air drying (HAD) is the predominant drying method employed in industry for FG. HAD is characterized by a rapid drying time, a high degree of automation, and straightforward operation [7,8], rendering it well-suited to the drying of large quantities of products. Negative-pressure circulating airflow-assisted desiccator drying (PCAD) is a proprietary non-thermal drying technology that combines low pressure and airflow with a desiccator to remove a portion of the oxygen from the drying process, thereby reducing the oxidation of the material. Compared with HAD, PCAD significantly reduces energy consumption. In addition, the desiccant used in PCAD can be recycled, further reducing drying costs and facilitating modern processing.

Root plants maintain strong activity for some time after harvest. In response to external environmental stimuli, the plant’s production of reactive oxygen species is increased, which can result in adverse effects on the plant. To mitigate this damage, enzymes within the plant work together to maintain homeostasis. During this process, metabolic changes occur, which can affect the quality of the plant [9,10]. At present, research on FG has been chiefly concerned with the analysis of its bioactive components and pharmacological effects. In contrast, literature employing metabolomics methods to address the impact of drying on FG quality remains comparatively sparse. Therefore, studying changes in metabolites within FG can provide critical insights into its quality under various drying methods. Widely targeted metabolomics analysis integrates the benefits of other metabolite detection techniques, rendering it efficaciously applicable to diverse food materials [11]. This helps to effectively and quantitatively determine the pathways related to the drying reaction mechanism of the sample [12], providing insights into the quality differences of FG caused by different drying methods.

At present, there are no reports on the mechanisms by which PCAD and HAD affect the types and contents of FG metabolites. Therefore, elucidating the metabolic changes of FG under different drying conditions holds practical significance. This study innovatively compared the FG enzyme activity of fresh and dried samples and made use of widely targeted metabolomics to identify FG metabolites. The aim was to explore the differential metabolites of FG under different drying treatments and propose the underlying mechanisms of metabolite changes in FG under different drying methods. The research results will aid in selecting appropriate drying methods to accommodate the industrial production of FG.

## 2. Materials and Methods

### 2.1. Reagents and Instrument

Reagents: Chromatographic grade methanol and acetonitrile, (Merck KGaA, Darmstadt, Germany); Chromatographic grade Formic acid, (Shanghai Aladdin Biochemical Technology Co., Ltd., Shanghai, China); superoxide dismutase (SOD) typing test kit, peroxidase (POD) and catalase (CAT) assay kit, (Nanjing Jiancheng Bioengineering Institute Co., Ltd., Nanjing, China). 3-hydroxy-3-methylglutaryl CoA reductase (HMGR) Elisa Kit, squalene synthase (SS) Elisa Kit and dammarenediol synthase (DS) Elisa Kit, (Andy Gene Biotechnology Co., Ltd., Beijing, China).

Instrumen: Negative pressure circulating drying system (self-made in the laboratory [13]), DHG-9030A Electrically Heated Blast Drying Oven (Shanghai Yiheng Technical Co., Ltd., Shanghai, China), HBS-ScanY Full-Wavelength Microplate Reader (NanJing DeTie Biotechnology Co., Ltd., Nanjing, China), MM 400 grinder (Retsch, Düsseldorf, Germany), VM200Vortex oscillator (Beijing Thmorgan Biotechnology Co., Ltd., Beijing, China), CBM30AUltra-performance liquid chromatograph (Shimadzu Corporation, Kyoto, Japan), 6500QTRAPTandem mass spectrometry (Applied Biosystems, Carlsbad, CA, USA).

### 2.2. Sample Collection and Drying Treatment

In August 2024, fresh forest ginseng samples were collected from the wild ginseng base in Huanren Manchu Autonomous County, Benxi City, Liaoning Province. The samples collected were then subjected to immediate analysis within the laboratory, with only those exhibiting optimal shape and integrity being selected for further experimentation. The moisture content of fresh forest ginseng is approximately 72%.

Two different drying methods were used to dry fresh forest ginseng (NP): the negative-pressure circulating airflow-assisted desiccator drying (PCAD) and hot air drying (HAD). In the PCAD method, FG is dried at room temperature (20–25 °C) for approximately 130.30 h until its moisture content is less than 12%. The specific method is as follows: the cleaned FG is evenly distributed in the drying chamber. The vacuum pump in the system is activated to extract air from the chamber, with the vacuum gauge reading 0.06 MPa. Desiccant silica gel is placed in the drying tube, and the circulation system is activated. The airflow velocity in the drying chamber is measured at 4.7 m/s [13]. In the HAD method, the cleaned FG is uniformly placed in a drying oven, the temperature is set to 65 °C for 4 h of drying, and the exhaust vent is opened to remove moisture, followed by drying at 40 °C. After drying for a total of 36.50 h, the moisture content of the HAD samples was less than 12%. Fresh and dried samples were promptly frozen in liquid nitrogen, pulverised, and maintained in a −80 °C freezer to be used subsequently. The samples were divided into three replicates, thus yielding a total of nine samples for each group.

### 2.3. Enzyme Activity Analyses [14]

Each group’s 0.5 g of powdered samples is to be placed in a pre-cooled mortar, blended into a slurry with 2.5 mL phosphate-buffered saline (pH 7.8), adjusted to a volume of 5 mL, centrifuged at 4000 rpm for 20 min at 4 °C, and the supernatant should be stored at 4 °C for later use. The determination of superoxide dismutase (SOD), peroxidase (POD), and catalase (CAT) enzymatic activity in FG was conducted using the hydroxylamine method, the colorimetric method, and the ammonium molybdate method, respectively. Enzyme activities of 3-hydroxy-3-methylglutaryl CoA reductase (HMGR), squalene synthase (SS), and dammarenediol synthase (DS) were measured using enzyme-linked immunosorbent assay kits.

### 2.4. Widely Targeted Metabolomic Analyses [15]

A freeze-drier was used to dry the samples, which were then ground into powder with a grinder (30 Hz, 1.5 min). After weighing 50 mg of the powder, 1200 μL of a 70% methanolic aqueous internal standard extract that had been precooled to −20 °C was added to the sample. For six repeats, the mixture was vortexed for 30 s every 30 min. The sample was centrifuged at 12,000 rpm for three minutes after vortexing, and the supernatant was carefully aspirated. The sample was moved to an injection vial for UPLC-MS/MS analysis utilizing the UPLC-ESI-MS/MS system (UPLC, ExionLC™ AD; MS) after being filtered via a 0.22 μm microporous membrane. An Agilent SB-C18 column (1.8 μm, 2.1 mm × 100 mm) was used for the analysis; the mobile phase consisted of acetonitrile and water with 0.1% formic acid (A and B, respectively); A gradient program using 95% A and 5% B as starting conditions was used to accomplish sample measurements. A linear gradient to 5% A and 95% B was programmed in 9 min, and for 1 min, the composition of 5% A and 95% B was maintained. A composition of 95% A and 5.0% B was then adjusted in 1.1 min and kept there for 2.9 min; the injection volume was 2 μL, and the column oven temperature was 40 °C. QTRAP-MS-ESI-triple quadrupole-linear ion trap was employed.

Ion spray voltage: 5500 V (positive ion mode)/−4500 V (negative ion mode); electrospray ionization temperature: 500 °C; With collision-induced ionization parameters set to high, the pressures of ion source gas I, gas II, and curtain gas are set to 50, 60, and 25 psi, respectively. A triple quadrupole mass spectrometer’s multiple reaction monitoring (MRM) mode is used to quantify metabolites. Set the nitrogen gas collision gas level to medium, and optimize the declustering potential and collision energy for each MRM ion pair through further optimization of the declustering potential and collision energy. A specific set of MRM ion pairs was monitored for each period based on the metabolites eluted during that period.

Specifically, to initially remove interference, the quadrupole in the MRM mode filters the target substance’s precursor ions, removing ions that correspond to other molecular weight substances. In the collision chamber, the precursor ions are caused to ionize before being disassembled into many fragment ions. The necessary characteristic fragment ion is then chosen by filtering the fragment ions through a triple quadrupole, which removes interference from non-target ions and improves repeatability and quantification. Based on the self-built MWDB (metware database), substance identification is performed using secondary spectrum information. During analysis, isotope signals, duplicate signals containing K^+^, Na^+^, and NH^4+^ ions, and duplicate signals from fragment ions that are themselves part of larger molecular weight substances are removed.

### 2.5. Data Analysis

Every analysis has been performed thrice. The analysis was conducted using SPSS 23.0 software (SPSS Inc., Chicago, IL, USA) for the analysis of variance (ANOVA) and Origin Lab software (Origin Lab Corp., Northampton, MA, USA) for graphical representation. The unsupervised principal component analysis (PCA) was conducted using the prcomp function in the R statistical software environment (https://www.r-project.org). The results of the hierarchical cluster analysis (HCA) of the samples and metabolites were displayed using heatmaps with dendrograms. The R package (Version 2.9.4) Complex Heatmap was utilised to perform the hierarchical clustering analysis (HCA). A colour spectrum was employed to represent the normalized signal intensities of the metabolites (unit variance scaling) for HCA. Log_2_FC absolute values (|Log_2_FC| ≥ 1.0 or ≤0.5) and variable importance in projection (VIP) scores (VIP ≥ 1) were utilised to identify differential metabolites for the two-group analysis. In addition to score plots and permutation plots created with the R package Metabo Analyst R, the VIP values were taken from the orthogonal partial least squares-discriminant analysis (OPLS-DA) results. Before the implementation of OPLS-DA, the data underwent a mean-centring and log transformation (log_2_). To prevent overfitting, a permutation test with 200 permutations was conducted. The KEGG Compound database (http://www.kegg.jp/kegg/compound/ accessed on 30 September 2024) was utilised for the annotation of the identified metabolites, and the KEGG Pathway database (http://www.kegg.jp/kegg/pathway.html accessed on 30 September 2024)) was subsequently mapped to the annotated metabolites.

## 3. Results

### 3.1. Enzyme Activity

**Figure 1:**
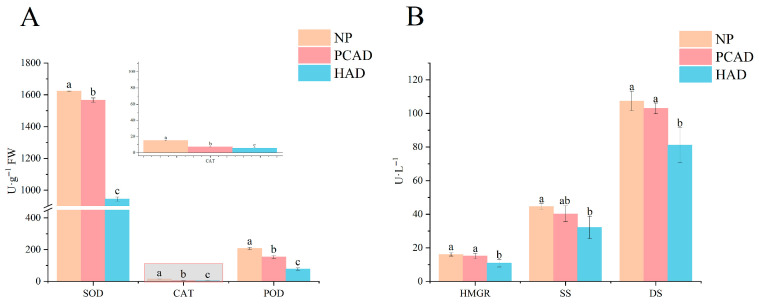
Enzyme activity. (**A**) Antioxidant enzyme activity; (**B**) Terpenoid synthesis-related enzymes. It is indicated by the utilisation of different letters (*p* < 0.05) that there are significant variations among the groups.

The enzyme activity of FG before and after drying is shown in Figure 1. A significant disparity in enzyme activity has been observed among the various groups under consideration. The activity of SOD, CAT, POD, HMGR, SS, and DS enzymes was highest in the NP sample, while the enzyme activity of FG decreased after drying, indicating that drying has a significant impact on enzymes, suggesting that FG experienced strong environmental stress stimuli during the drying process. Additionally, the FG enzyme activity also showed significant changes under different drying methods, with the antioxidant enzyme activity in PCAD being higher than in HAD, suggesting that PCAD may more effectively activate the antioxidant enzyme system of FG, placing FG in a more active metabolic process, thereby facilitating metabolic activities. The enzymes involved in terpenoid synthesis determine, to a great extent, the level at which ginsenoside synthesis occurs. The HMGR and SS enzyme activities in PCAD were higher than those in HAD, indicating that PCAD is more conducive to the production of 2,3-oxo-squalene, which is required for the subsequent synthesis of triterpenoid compounds, thereby promoting the biosynthesis of ginsenosides. Additionally, PCAD retains more DS activity, indicating that the synthesis activity of dammarane-type ginsenosides is enhanced in FG.

### 3.2. Metabolome Analysis

#### 3.2.1. PCA and OPLS-DA Analysis Results

**Figure 2:**
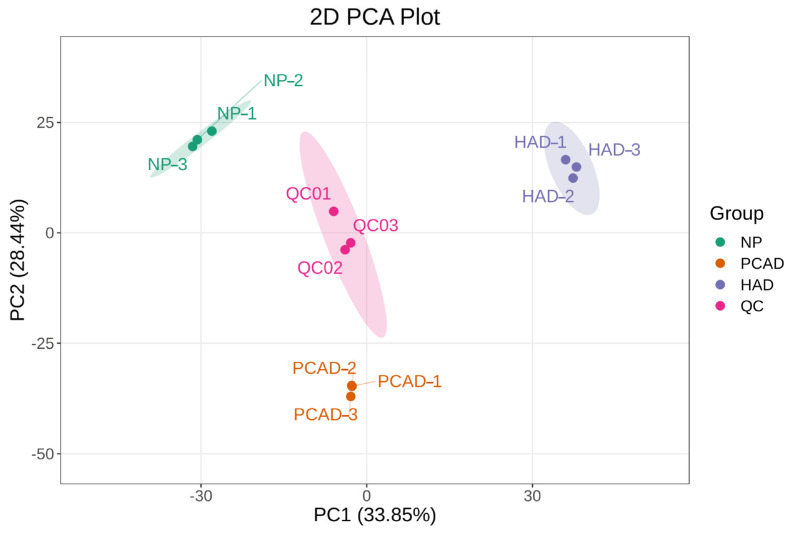
Results of PCA.

PCA is a statistical technique that can be used to reveal the internal structure among several variables, with the use of a reduced number of principal components. Therefore, PCA analysis can be used to understand the overall differences among grouped subjects and the variation present within each group. Figure 2 displays the PCA score plots for the study’s quality control (QC) samples and sample data from each group. The QC samples and metabolites within each treatment group are relatively clustered, indicating that metabolites within groups are relatively concentrated and exhibit minimal variability. However, the distribution of metabolites in FG treated with different drying methods is relatively dispersed, indicating that the metabolic profiles of FG vary significantly under different drying conditions, suggesting a strong correlation between drying methods and alterations to metabolites within FG.

**Figure 3:**
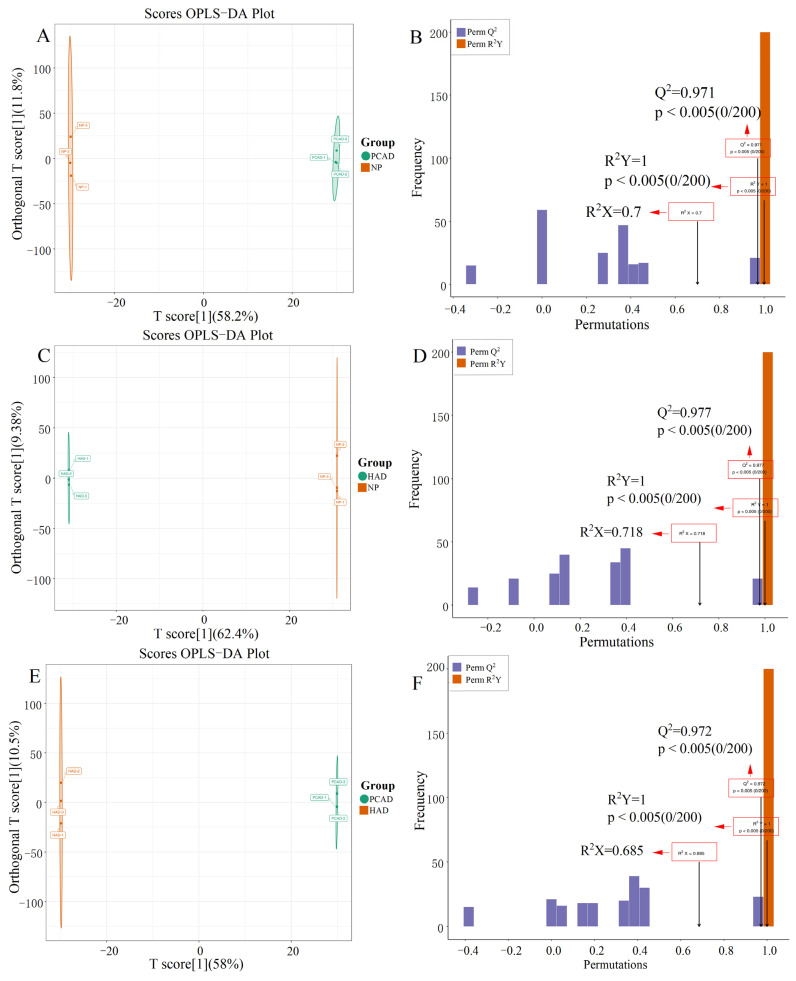
Results of OPLS-DA. OPLS-DA score chart (**A**,**C**,**E**) and verification chart (**B**,**D**,**F**). (**A**,**B**) PCAD vs. NP, (**C**,**D**) HAD vs. NP, and (**E**,**F**) PCAD vs. HAD.

Furthermore, the OPLS-DA analyses yielded comparable outcomes to those of the PCA analyses (Figure 3). The NP and PCAD exhibited high predictability (Q^2^) and strong goodness-of-fit (R^2^X, R^2^Y) (Q^2^ = 0.971, R^2^X = 0.7, R^2^Y = 1, Figure 3A,B), as did the NP and HAD (Q^2^ = 0.977, R^2^X = 0.718, R^2^Y = 1, Figure 3C,D) and the HAD and PCAD (Q^2^ = 0.972, R^2^X = 0.685, R^2^Y = 1, Figure 3E,F). These results support the validity of the OPLS-DA model for screening of different metabolites by showing that it exhibits stability and high reliability for this dataset.

#### 3.2.2. Composition and Classification of Metabolites

**Figure 4:**
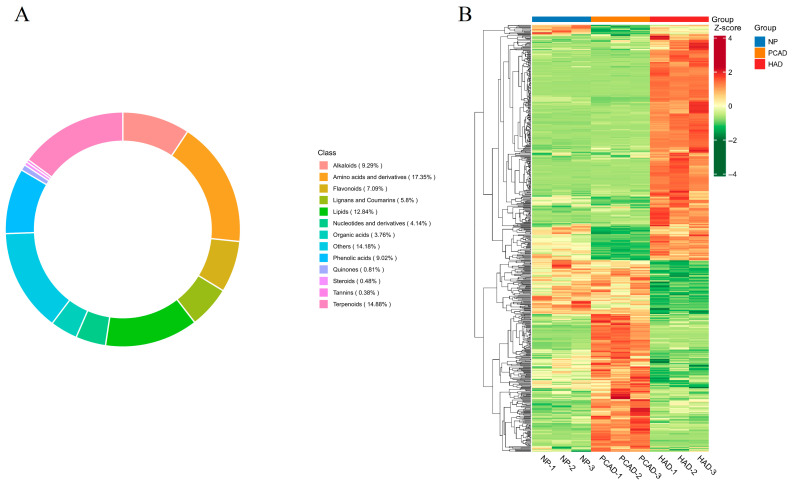
Composition and Classification of Metabolites. (**A**) Metabolite type ring diagram; (**B**) Cluster analysis.

A total of 1862 metabolites from NP, PCAD, and HAD were found using widely targeted metabolomics analysis techniques (Table S1). As shown in Figure 4A, Amino acids and derivatives (17.35%), terpenoids (14.88%), lipids (12.84%), alkaloids (9.29%), phenolic acids (9.02%), flavonoids (7.09%), lignans and coumarins (5.80%), nucleotides and derivatives (4.14%), organic acids (3.76%), quinones (0.81%), steroids (0.48%), tannins (0.38%) and other (14.18%) were the 13 categories into which these compounds were arranged in order of total compounds. Terpenoids, phenolic acids, and flavonoids were the most common secondary metabolites in FG, while tannins and steroids were found in smaller amounts.

HCA can classify individuals or samples based on their characteristics. The HCA results show the different metabolites that are significantly different between the groups, as well as the differences between these metabolites. The three groups of samples are all similar (Figure 4B). It can be observed that metabolites in fresh and differently dried samples exhibit significant differences, clearly divided into three clusters, indicating that different drying methods have a significant impact on the types and levels of metabolites.

The MRM mode was used to quantify the metabolites, and the mass spectrometry signal intensity was used to determine their abundances. Metabolites were quantitatively compared by category, and ginsenosides and sesquiterpene metabolites in FG under different drying methods were compared. Additionally, the specific details of the qualitative analysis of ginsenosides and sesquiterpenes are shown in Table S2. A total of 38 ginsenosides, including common ones like Rg_1_, Re, Rf, Rb_1_, Rc, Rb_2_, and Rd, were found in PCAD and HAD, as indicated in Table 1. Rare ginsenosides like Rg_3_ and Rh_2_ have been identified in the groups in addition to common ginsenosides. The total ginsenoside content (sum of individual ginsenosides) showed PCAD > HAD, indicating that PCAD retained more ginsenosides than HAD, with PCAD ginsenoside content increased by 1.98% compared to HAD.

The results for sesquiterpenoid compounds are shown in Table 2, with a total of 44 sesquiterpenoids detected in both PCAD and HAD. The total sesquiterpene content (sum of sesquiterpene content) shows that PCAD > HAD, with the total sesquiterpene content in PCAD increased by 11.41% compared to HAD, indicating that different drying methods affect sesquiterpenes, possibly related to temperature, oxygen, and degree of sealing.

#### 3.2.3. Differential Metabolites (DMs)

**Figure 5:**
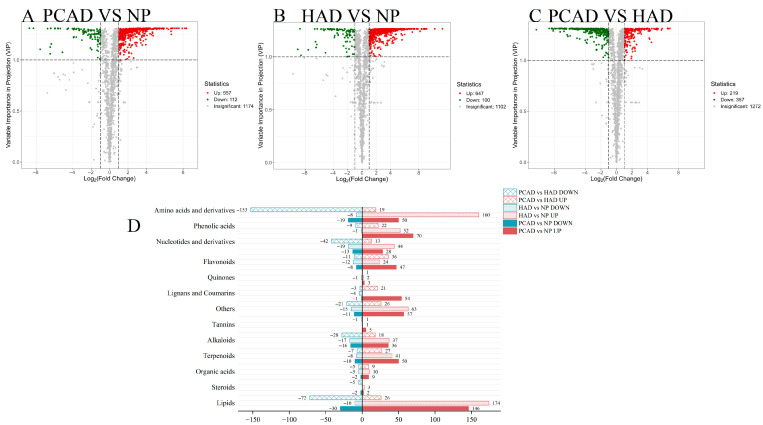
DMs analysis. (**A**–**C**) Volcano plots. Gray dots indicate metabolites that are not substantially stated, red dots indicate metabolites that are upregulated, and green spots indicate metabolites that are downregulated. (**D**) The classification of differential metabolites. Differentially expressed metabolites that are elevated are shown in red columns, and those that are downregulated are shown in blue columns. The number of metabolites that have different expressions across every pairwise comparison of FG is indicated by the numbers on the side of the columns.

Figure 5 displays the findings of the volcano plot for DMs. There were notable differences in the quantity and kinds of DMs between the fresh and dry comparison groups. There were 669 DMs between PCAD and NP. Compared with NP as the control group, 557 metabolites were significantly increased in PCAD, and 112 metabolites were significantly decreased in PCAD (as shown in Figure 5A); There were 747 DMs between HAD and NP. As illustrated in Figure 5B, there was a significant rise in 647 metabolites and a significant drop in 100 metabolites in HAD when compared to NP, the control group. There were 576 differences in DMs between PCAD and HAD across the various drying comparison groups. As seen in Figure 5C, there was a significant increase in 219 metabolites and a significant decrease in 357 metabolites in PCAD when compared to the HAD group. This suggests that temperature has a significant impact on metabolites.

Further classification and comparison of the DMs produced by fresh and different drying methods in FG were conducted. Except for the “other” category, the DMs fall into 12 different categories, as seen in Figure 5D. They are mostly found in lipids, amino acids and derivatives, phenolic acids, terpenoids, lignin, and coumarins. Pairwise comparisons between fresh and dried samples (PCAD vs. NP and HAD vs. NP) revealed that, except for lignin and coumarins, the amount of upregulated DMs within PCAD and HAD was larger than that at the corresponding NP, suggesting that drying may activate or inhibit certain physiological and metabolic activities. In the paired comparison of the drying groups (PCAD vs. HAD), the number of secondary metabolites detected, including organic acids, terpenoids, lignin and coumarins, flavonoids, and phenolic acids, was significantly greater than the amount of downregulated metabolites, showing that PCAD significantly upregulates secondary metabolites in FG compared to HAD.

**Figure 6:**
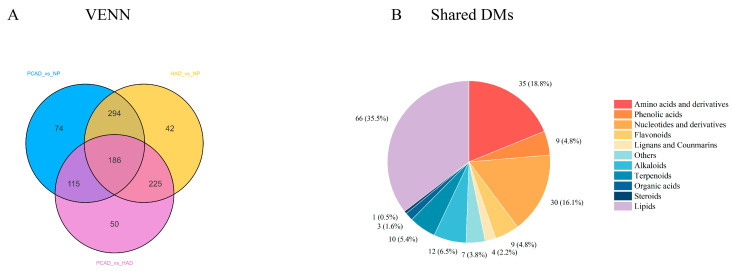
Venn diagram and shared DMs pie chart. (**A**) Venn diagram of differential metabolites; (**B**) Classification of the 186 shared DMs.

Analyzing the impact of various drying techniques on FG metabolites is made easier by the identification of DMs. All groups were screened, and 986 DMs were found. The distinct and shared DMs among the PCAD vs. NP, HAD vs. NP, and PCAD vs. HAD groups were shown using a Venn diagram. In the PCAD vs. NP group, the HAD vs. NP group, and the PCAD vs. HAD group, 74, 42, and 50 distinct DMs were found, respectively, as illustrated in Figure 6A. The 186 shared DMs, which are regarded as important metabolites between NP, PCAD, and HAD, are also described in Figure 6B. Lipids, amino acids and derivatives, nucleotides and derivatives, alkaloids, terpenoids, and phenolic acids are the main components of these DMs.

#### 3.2.4. Metabolic Pathway Analysis

**Figure 7:**
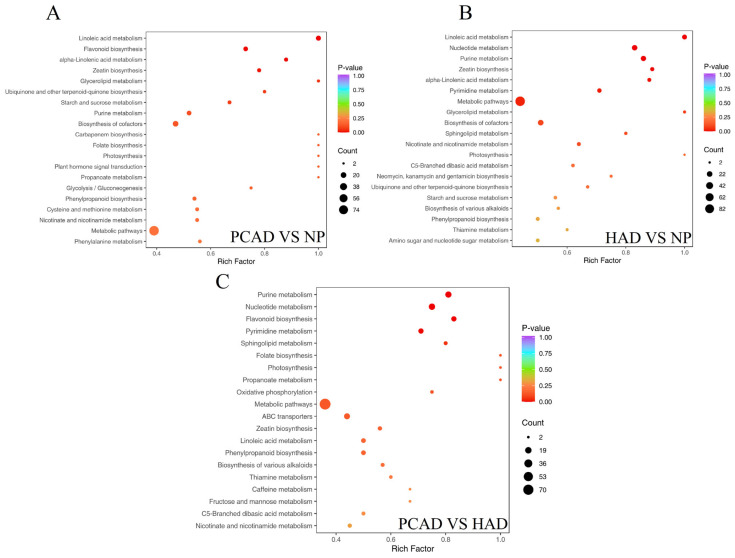
Metabolomic enrichment pathway analysis. (**A**) PCAD vs. NP, (**B**) HAD vs. NP and (**C**) PCAD vs. HAD.

Different metabolic alterations accompany the drying procedure for FG using various drying techniques. The Kyoto Encyclopedia of Genes and Genomes database was used to import the examined DMs to clarify these intricate metabolic change networks. Figure 7 displays the relevant metabolic pathways based on KEGG annotation and enrichment results. The top 20 KEGG-enriched metabolic pathways are displayed in the bubble chart.

In the PCAD vs. NP group (Figure 7A), linoleic acid metabolism showed the most significant enrichment (*p* < 0.001). Other significantly enriched pathways include flavonoid biosynthesis and α-linolenic acid metabolism (*p* < 0.01) and zeatin biosynthesis (*p* < 0.05). In the HAD vs. NP group (Figure 7B), linoleic acid metabolism, nucleotide metabolism, and purine metabolism showed the most significant enrichment (*p* < 0.001). Other significantly enriched pathways included zeatin biosynthesis and α-linolenic acid metabolism (*p* < 0.01), as well as pyrimidine metabolism and metabolic pathways (*p* < 0.05). In the PCAD vs. HAD group (Figure 7C), purine metabolism, nucleotide metabolism, and flavonoid biosynthesis showed the most significant enrichment (*p* < 0.001). Pyrimidine metabolism (*p* < 0.01) and sphingolipid metabolism (*p* < 0.05) were two more pathways that were considerably enriched.

Linoleic acid metabolism, nucleotide metabolism, purine metabolism, zeatin biosynthesis, α-linolenic acid metabolism, pyrimidine metabolism, and flavonoid biosynthesis were the metabolic pathways that were significantly enriched and shared by the three comparison groups. In total, the seven pathways mentioned above contained key pathway metabolites, which reflect the likely mechanisms of the impacts on FG metabolites after drying; therefore, different drying methods regulate FG quality by changing the relevant DMs for these elements’ pathways.

#### 3.2.5. Analysis of Metabolic Pathways of Representative Bioactive Ingredients

**Figure 8:**
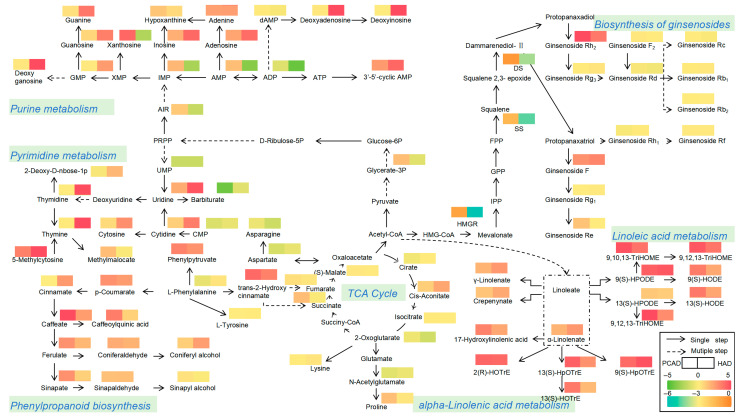
Changes of metabolites in different drying methods—transformation pathways.

Several representative classes of terpenes, lipids, nucleotides, and phenolic acids were chosen for additional examination of the change-transformation network by integrating the KEGG pathway results to correlate various metabolite kinds through connecting their biosynthetic pathways. This was done to further investigate the effects of various drying techniques on FG metabolites (Figure 8).

The citrate cycle had an enrichment of seven organic acid metabolites. Succinate and 2-oxoglutarate were accumulated at higher levels in PCAD samples, while cis-Aconitate was accumulated at higher levels in HAD samples, demonstrating that different drying methods affect the citrate cycle.

Ginsenosides are synthesized via the mevalonate (MVA) pathway and then converted into terpenoids using Acetyl-CoA as a precursor and catalyzed by related enzymes. According to the findings, the activities of the three terpenoid synthesis-related enzymes reduced significantly after drying, but the residual enzyme activities in PCAD were significantly higher than those in HAD. The Figure shows that ginsenoside Rh_2_, ginsenoside F, and ginsenoside Re accumulate significantly in the ginsenoside biosynthetic pathway, with PCAD having significantly greater levels of ginsenoside Rh_2_ and ginsenoside Re than HAD. These findings suggested that the increased enzyme activity of terpenoid synthesis in PCAD could boost the synthesis of ginsenosides.

Acetyl-CoA indirectly promotes lipid metabolism through linoleic acid and α-linolenic acid metabolism, which are linked to lipoxide formation in fatty acid metabolism. According to the figure, fatty acids were considerably accumulated in both drying methods, and 15 lipids have been enhanced in the metabolism of linoleic acid and α-linolenic acid. This implies that lipid metabolism is enhanced by drying.

Purine metabolism involved 17 nucleotides and derivatives, with only 6 DMs accumulating at high levels in PCAD samples, particularly Xanthosine; 9 DMs accumulating at high levels in HAD samples; and 2 nucleotides and derivatives that were not significantly different in the two drying methods. Ten nucleotides and derivatives were found to be concentrated in pyrimidine metabolism, with 7 of these metabolites considerably accumulating in HAD. Purine and pyrimidine metabolism studies showed that HAD enhances the formation of nucleotides and derivatives, which could be temperature-dependant.

L-Phenylalanine content was much lower in PCAD than in HAD, although phenylpyruvate, trans-2-hydroxy cinnamate, and downstream phenylpropanoid biosynthesis were significantly higher, indicating that PCAD promotes the generation of other metabolites from phenylalanine. 10 DMs were enriched in phenylpropanoid biosynthesis, including 1 aldehyde, 2 alcohols, and 7 phenolic acids. Only 2 DMs accumulated more in HAD, whereas caffeate, caffeoylquinic acid, ferulate, and sinapate were significantly more retained in PCAD than in HAD. The findings indicate that PCAD enhances phenylpropanoid biosynthesis more than HAD, implying that PCAD also promotes the formation of flavonoids, lignans, and coumarins, which generates more secondary metabolites. In conclusion, the 2 drying methods may promote the production of primary and secondary metabolites, respectively, due to their drying characteristics.

## 4. Discussion

### 4.1. Effect of Drying on Enzyme Activity

The enzyme activities were significantly reduced following drying, suggesting that the loss of water in the tissues significantly affects the enzyme activities in FG. The plant body creates a lot of reactive oxygen radicals, which harm the organism when it is exposed to adverse conditions like drying and water loss. To preserve cellular homeostasis and reduce this harm this damage, the plant body’s SOD, CAT, and POD cooperate to transform harmful reactive oxygen radicals into harmless oxygen and water [16,17]. Enzyme activation stimulates a variety of metabolic activities in the plant, making the synthesis and transformation of plant secondary metabolites more favorable. It has been proven that when the postharvest drying temperature is relatively low, the buildup of metabolites in the plant is mostly mediated by the sustained enzyme response to water stress. When drying temperatures are relatively high, enzyme activity is gradually reduced or inactivated, chemically driven reactions take precedence, and more highly oxidized components are generated [18]. At room temperatures, PCAD causes a flat-water loss slowly, which extends the duration of enzymatic activity. After drying, enzymatic activity was much lower than in NP, while PCAD retained greater activity than HAD. It also implies that PCAD can produce additional secondary metabolites. Since temperature directly influences the conclusion period during the enzyme response and the course of the thermochemical reaction, which leads to the synthesis of DMs of different types and contents and eventually affects the quality of the FG, it is evident that temperature plays a crucial role in controlling the drying process [19].

### 4.2. Effects of Drying on Metabolome

Generally, temperature, water, and external pressures are the main elements that affect drying-induced changes in active components, and they also reveal the mechanics behind the chemical processes that take place during drying [20]. Chemical induction caused by heat degradation, as well as continuous catalysis by enzymes in response to drought stress, may be the primary drivers of metabolite differences across 2 different FG drying procedures. This study examined 5 metabolites that alter the quality of FG.

With a variety of pharmacological actions, including immunological stimulation, antitumor activity, and anti-aging characteristics, ginsenosides are significant terpenoid active components in FG [21]. Metabolomic data showed that ginsenosides decreased more after HAD drying, which may be due to the temperature (40–65 °C) used in the HAD drying treatment of FG, which is within the active period of enzymes, causing enzymes to break down more ginsenosides. Furthermore, the decreased saponin concentration in HAD may be temperature dependent [22]. FG contains thermally unstable malonyl ginsenosides [23], which disintegrate into other components. Li et al. discovered that by drying at a lower temperature, more ginsenoside content could be maintained [24]. PCAD does not entail temperature variations during drying and is carried out in an airtight environment, which can help to minimize oxidation somewhat. Therefore, PCAD retained greater total ginsenoside content than HAD.

Lipids are one of the primary metabolic components in FG, according to metabolomics studies. Most lipid metabolites showed a significant increase in total content after drying, with a notable accumulation under HAD drying conditions. This may be related to the fact that changes in fatty acid-related metabolites often occur at temperatures between 45–60 °C [18]. It is generally understood that lipid oxidation in high-temperature and high-oxygen settings is a primary cause of poor food quality. PCAD, unlike HAD, can inhibit lipid oxidation. On the one hand, PCAD is carried out at room temperature. Ambient temperature keeps cells inactive and makes their walls less susceptible to disturbance, potentially reducing lipid release. On the other hand, PCAD lowers FG’s prolonged exposure to air. Thus, the choice of drying method used can have a substantial impact on the lipid content and, as a result, the quality of FG.

A number of the main secondary metabolites that plants produce under biotic or abiotic stressors is phenylpropanes, and their biosynthesis pathway generates a variety of antioxidants, such as phenolics, lignans, and flavonoids, to protect the plant from harm. Phenylpropanoid biosynthesis was significantly increased in PCAD in the current investigation. The activation of the phenylalanine pathway, which supplies precursors for the development of flavonoids, lignans, and coumarins, is confirmed by the rise in phenylalanine metabolites and derivatives, including cinnamate, p-coumarate, and 2-hydroxycinnamate. Moreover, phenylalanine ammonia-lyase (PAL) is the main enzyme in the enzymatic reaction that produces phenylpropanoid biosynthesis. Plant PAL expression is normally low and usually rises in response to biotic or abiotic stressors [25]. Therefore, it seems sense to assume that PAL gets started after FG dry and becomes more active in PCAD, raising the concentration of lignin and coumarin and stimulating phenylalanine metabolism.

Amino acid production is essential for plant growth and stress tolerance [26]. During desiccation, considerable buildup of amino acids and derivatives modifies plant defense against drought stress through osmotic homeostasis and maintenance of the stability of cell membrane structures. Amino acid side chains can be altered by heat treatment, and the degree of the change increases as the temperature rises [27], producing a range of oxidative products. The results of the study showed that the HAD relative concentration of amino acid metabolites increased significantly in comparison to the PCAD, suggesting that heating significantly impacted amino acid metabolism during FG drying.

Plant growth, development, metabolism, and substance synthesis are all greatly aided by nucleotides, which are vital cellular constituents [28]. Precursor materials needed for downstream synthesis are produced through the metabolism of purines and pyrimidines. Particularly crucial resources for the synthesis of nucleotides are the purine and pyrimidine bases produced during purine and pyrimidine metabolism. HAD aided purine and pyrimidine metabolism in this investigation. This promotion may be connected to temperature, as heating causes ATP and GTP breakdown. The study found that HAD had considerably higher levels of Adenosine, Guanosine, Deoxyguanosine, Deoxyinosine and Deoxyadenosine, which are breakdown products of ATP and GTP, than PCAD. These increases eventually led to an increase in purine metabolism, and so nucleotide production.

## 5. Conclusions

Drying methods significantly influence the enzymatic activity and metabolic profile of FG, potentially due to chemical induction resulting from thermal degradation and the sustained catalytic response of enzymes under drought stress. In terms of enzymatic activity, water loss during drying leads to a significant reduction in enzyme activity in dried FG compared to fresh samples. However, PCAD better preserves antioxidant enzymes and enzymes involved in terpenoid synthesis than HAD, suggesting that PCAD may be more favorable for maintaining metabolic activity. Widely targeted metabolomic analysis identified 13 classes of metabolites, including amino acids, lipids, nucleotides, terpenoids, and phenolic acids, etc., and further identified 986 differential metabolites. Pairwise comparisons indicated that HAD promotes the accumulation of primary metabolites, whereas PCAD enhances the production of secondary metabolites. By integrating metabolite profiles and their associated biosynthetic pathways under different drying methods, it was found that these pathways are predominantly linked to the MVA pathway, lipid metabolism, phenylpropanoid biosynthesis, and nucleotide metabolism. These findings reveal the potential mechanisms underlying metabolite changes in FG under various drying conditions, contributing to a better understanding of how drying treatments affect FG quality from a metabolic perspective. The findings from this research will provide valuable insights into the drying processes applicable to fresh FG while offering scientific foundations and technical support for selecting appropriate drying methodologies.

## Figures and Tables

**Table 1:** Ginsenosides in metabolites of forest ginseng.

Component	Average Relative Content	
	PCAD	HAD
Ginseng Rh_4_	2.11 × 10^5^	2.22 × 10^5^
Ginseng Rg_7_	5.21 × 10^5^	6.17 × 10^5^
Ginseng Rc	2.32 × 10^5^	1.80 × 10^5^
Ginseng Rh_14_	1.15 × 10^6^	9.70 × 10^5^
Ginseng Rk_1_	3.20 × 10^5^	4.52 × 10^5^
Ginseng F_4_	3.20 × 10^5^	4.52 × 10^5^
Ginseng F_3_	2.05 × 10^4^	2.68 × 10^4^
Pseudo ginsenoside F_11_	6.67 × 10^4^	5.51 × 10^4^
Ginseng Re_5_	3.26 × 10^5^	3.11 × 10^5^
Ginseng Rg_6_	7.56 × 10^5^	7.25 × 10^5^
Ginseng Rd	1.66 × 10^5^	1.51 × 10^5^
Malonyl ginsenoside Re	1.71 × 10^5^	6.69 × 10^4^
Ginseng Rh_15_	4.92 × 10^5^	4.70 × 10^5^
Ginseng Rh_8_	1.57 × 10^5^	2.28 × 10^5^
Malonyl ginsenoside Rb_1_	6.75 × 10^5^	8.65 × 10^5^
Ginseng Rh_19_	8.13 × 10^4^	1.17 × 10^5^
Ginseng Rd_2_	5.58 × 10^4^	3.58 × 10^4^
Ginseng Mx	4.88 × 10^5^	4.35 × 10^5^
Ginseng Rf_1_	1.66 × 10^4^	1.10 × 10^4^
Ginseng Rh_2_	1.15 × 10^5^	1.94 × 10^4^
Ginseng Rs_1_	1.32 × 10^4^	1.96 × 10^4^
Malonyl ginsenoside Rd	2.33 × 10^5^	1.84 × 10^5^
Ginsenoside Rk_2_-acetyl	2.78 × 10^4^	3.69 × 10^4^
Ginseng ST_2_	1.08 × 10^4^	1.17 × 10^3^
Ginseng Ro	1.61 × 10^6^	1.69 × 10^6^
Ginseng Rb_1_	1.35 × 10^5^	1.85 × 10^5^
Ginseng Rg_2_	1.53 × 10^5^	1.61 × 10^5^
Ginseng F_2_	1.53 × 10^4^	1.30 × 10^4^
Ginseng Rf	9.93 × 10^5^	9.84 × 10^5^
6′-O-acetyl-Ginsenoside Rg_1_	3.55 × 10^6^	3.33 × 10^6^
5,6-didehydroginsenoside Rd	4.32 × 10^5^	3.54 × 10^5^
Ginseng Rg_1_	2.06 × 10^6^	2.17 × 10^6^
Ginseng La	1.15 × 10^6^	9.70 × 10^5^
Ginseng Re	1.41 × 10^5^	1.03 × 10^5^
Ginseng Rb_2_	1.03 × 10^5^	1.19 × 10^5^
Ginseng Rg_3_	5.31 × 10^3^	7.32 × 10^3^
Ginseng Rh_1_	8.79 × 10^3^	1.02 × 10^4^
Ginseng F_1_	3.81 × 10^3^	5.06 × 10^3^
Total ginsenosides	1.72 × 10^7^	1.68 × 10^7^

**Table 2:** Sesquiterpenoids in metabolites of forest ginseng.

Component	Average Relative Content	
	PCAD	HAD
JiangxiBaiyingsu I	3.42 × 10^5^	5.27 × 10^5^
3,4-Dihydroxy-7,8-dihydro-β-ionone-4-O-β-D-glucoside	8.74 × 10^5^	6.49 × 10^5^
polygodial	1.58 × 10^5^	1.46 × 10^5^
1,6-Dihydroxy-4(14)-eudesmene	5.13 × 10^4^	4.85 × 10^4^
β-Eudesmol	1.30 × 10^4^	1.54 × 10^4^
3,7,11-trimethyldodeca-3,7-diene-1,10,11-triol 1-O-(beta-D-Xylopyranosyl)-beta-D-glucopyranoside	2.14 × 10^5^	1.89 × 10^5^
Humulene epoxide II	1.75 × 10^5^	1.58 × 10^5^
Cyclocolorenone	9.96 × 10^4^	6.86 × 10^4^
Anhydro-β-rotunol	3.62 × 10^5^	2.97 × 10^5^
Septemlobin D	6.29 × 10^4^	7.75 × 10^4^
6α,10α-Dihydroxy-1-oxoeremophila-7(11),8(9)- dien-12,8-olide	8.87 × 10^6^	8.79 × 10^6^
Nootkatone	7.58 × 10^4^	8.53 × 10^4^
3-Hydroxy-3,7,11-trimethyldodeca-1,6E,10-trien- 9-yl isobutyrate	2.40 × 10^6^	2.30 × 10^6^
13-Hydroxygermacrone	1.23 × 10^5^	1.07 × 10^5^
Aristolone	6.45 × 10^4^	3.10 × 10^4^
3,7,11-trimethyldodeca-3,7-diene-1,10,11-triol 10-O-(beta-D-Xylopyranosyl)-beta-D-glucopyranoside	1.16 × 10^5^	1.73 × 10^5^
Sessilifol O	1.84 × 10^4^	2.33 × 10^4^
Leucodin	6.45 × 10^4^	3.47 × 10^4^
(6R,9R)-3-Oxo-α-ionol-β-D-malonyl-glucoside	5.43 × 10^4^	2.04 × 10^4^
Micheliolide	1.81 × 10^4^	1.01 × 10^4^
Septemlobin E	4.28 × 10^4^	4.54 × 10^4^
3-Oxo-Alpha-Ionol diglucoside	7.64 × 10^4^	6.31 × 10^4^
solajiangxins H	4.47 × 10^4^	3.07 × 10^4^
Santalol A	3.71 × 10^5^	3.25 × 10^5^
8-methoxy-3,4,5-trimethyl-5,6,7,8-tetrahydrobenzo[f][1]benzofuran	1.30 × 10^5^	6.23 × 10^4^
11,12-O-Isopropyfidenesolajiangxin F	5.03 × 10^4^	4.99 × 10^4^
Blumenol B malonyl Glucoside	6.03 × 10^4^	1.60 × 10^4^
Blumenol C malonyl Glucoside	2.16 × 10^4^	1.90 × 10^4^
Dendronobilin I	4.58 × 10^5^	8.93 × 10^4^
10α-Hydroperoxy-guaia-1,11-diene	3.71 × 10^4^	2.64 × 10^4^
Icariside B2	1.19 × 10^5^	9.97 × 10^4^
1,1′-(6-hydroxy-2,3-dihydro-1H-indene- 2,5-diyl)bis(ethan-1-one)	1.30 × 10^5^	1.11 × 10^5^
Glucosyl dihydroroseoside	3.13 × 10^4^	2.06 × 10^4^
15-Hydroxysessilifol F	2.89 × 10^4^	3.56 × 10^4^
(2E)-3-(1-Hydroxy-2,6,6-trimethyl-4-oxo-2-cyclohexen-1-yl)-2-propenoic acid	5.39 × 10^4^	2.27 × 10^4^
Ampelopsisionoside	8.56 × 10^5^	5.14 × 10^5^
Dihydrophaseic acid	4.70 × 10^4^	1.14 × 10^4^
Cryptomeridiol	8.21 × 10^4^	9.27 × 10^4^
5-Hydroxylbisabolon-9-one	5.16 × 10^4^	4.53 × 10^4^
Glucosyl 10,11-dihydroxy-2,6-farneSadienoate	2.08 × 10^5^	1.83 × 10^5^
patrinioside	2.74 × 10^6^	2.17 × 10^6^
6′-O-Glucosylaucubin	1.26 × 10^5^	2.35 × 10^5^
Dihydrovomifoliol-O-β-D-glucoside	8.74 × 10^5^	6.49 × 10^5^
Abscisic acid	1.31 × 10^4^	7.61 × 10^3^
Total sesquiterpenoids	2.08 × 10^7^	1.87 × 10^7^

## Data Availability

The data that support the findings of this study are available from the corresponding author upon reasonable request.

## Supplementary Materials

The following supporting information can be downloaded at: https://www.mdpi.com/article/10.3390/foods14152753/s1, Table S1: The result of metabolites; Table S2: Qualitative analysis of some important metabolites.

## References

[1] Xu X.-F., Cheng X.-L., Lin Q.-H., Li S.-S., Jia Z., Han T., Lin R.-C., Wang D., Wei F., Li X.-R. (2016). Identification of mountain-cultivated ginseng and cultivated ginseng using UPLC/oa-TOF MSE with a multivariate statistical sample-profiling strategy. J. Ginseng Res..

[2] Zhu L.L., Luan X.N., Yuan Y., Dou D.Q., Huang L.Q. (2021). The characteristics of ginsenosides and oligosaccharides in mountain- and garden-cultivated ginseng. J. Sci. Food Agric..

[3] Xu X., Yu S., Dou D. (2014). Comparison of the effects of garden ginseng on immune function in mice. Ginseng Res..

[4] Huang J., Liu D., Wang Y., Liu L., Li J., Yuan J., Jiang Z., Jiang Z., Hsiao W.W., Liu H. (2022). Ginseng polysaccharides alter the gut microbiota and kynurenine/tryptophan ratio, potentiating the antitumour effect of antiprogrammed cell death 1/programmed cell death ligand 1 (anti-PD-1/PD-L1) immunotherapy. Gut.

[5] Liu D., Li Y., Xu H., Sun S., Wang Z. (2008). Differentiation of the Root of Cultivated Ginseng, Mountain Cultivated Ginseng and Mountain Wild Ginseng Using FT-IR and Two-Dimensional Correlation IR Spectroscopy. J. Mol. Struct..

[6] Thamkaew G., Sjöholm I., Galindo F.G. (2020). A review of drying methods for improving the quality of dried herbs. Crit. Rev. Food Sci..

[7] Das I., Arora A. (2018). Alternate microwave and convective hot air application for rapid mushroom drying. J. Food Eng..

[8] Jalali N., Goli M., Sheikhan N., Shahi S., Kermani S. (2024). Effect of Calcium Chloride Extracted from Poultry Eggshells on the Physicochemical Properties of Iranian High-Fat White Cheese. Iran. J. Chem. Chem. Eng..

[9] Zhang S., Yu Q.Y., Niu L.C., Yuan H.B., Shan X.J., Hua J.J., Chen L., Zhang Q.T., Feng Y.N., Yu X.L. (2025). Integration of intelligent sensory evaluation, metabolomics, quantification, and enzyme activity analysis to elucidate the influence of first-drying methods on the flavor formation of congou black tea and its underlying mechanism. Food Chem..

[10] Xu P., Su H., Zhao S., Jin R., Cheng H., Xu A., Lai W., Yin X., Wang Y. (2020). Transcriptome and Phytochemical Analysis Reveals the Alteration of Plant Hormones, Characteristic Metabolites, and Related Gene Expression in Tea (*Camellia sinensis* L.) Leaves During Withering. Plants.

[11] Zhang H.F., Lu Q., Liu R. (2022). Widely targeted metabolomics reveals the effect of fermentation on the chemical composition of bee pollen. Food Chem..

[12] Yang M., Yang J., Su L., Sun K., Li D., Liu Y., Wang H., Chen Z., Guo T. (2019). Metabolic profile analysis and identification of key metabolites during rice seed germination under low-temperature stress. Plant Sci..

[13] Cai E., Du R., Han J., Dang W., Zhao Y., Xue L. (2024). A Kind of Negative Pressure Dry Processing Internal Circulation System. China ZL.

[14] Ma R., Sun L., Chen X., Mei B., Chang G., Wang M., Zhao D. (2016). Proteomic analyses provide novel Insights into plant growth and ginsenoside biosynthesis in forest cultivated *Panax ginseng* (F. Ginseng). Front. Plant Sci..

[15] Xiao J.Q., Gu C.Q., He S., Zhu D.X., Huang Y.K., Zhou Q.Q. (2021). Widely targeted metabolomics analysis reveals new biomarkers and mechanistic insights on chestnut (*Castanea mollissima* Bl.) calcification process. Food Res. Int..

[16] Li D., Zhang X., Li L., Aghdam M., Wei X., Liu J., Xu Y., Luo Z. (2019). Elevated CO_2_ delayed the chlorophyll degradation and anthocyanin accumulation in postharvest strawberry fruit. Food Chem..

[17] Li T., Shi D., Wu Q., Zhang Z., Qu H., Jiang Y. (2019). Sodium para-aminosalicylate delays pericarp browning of litchi fruit by inhibiting ROS-mediated senescence during postharvest storage. Food Chem..

[18] Pratama B.P., Pranoto Y., Supriyadi S., Swasono R.T. (2022). Effect of drying time and temperature to the chemical properties and enzymatic activities related to the β-ocimene production in syzygium polyanthum leaves. Trends Sci..

[19] Wang J., Yuan H., Hua J., Jiang Y., Dong C., Deng Y., Yang Y. (2020). Effects of second-drying process parameters on the hot-air drying characteristics and quality of congou black tea. Transact. Chin. Soc. Agr. Eng..

[20] Wang Y., Zhang J., Wang Z., Cui F., Zhang Q., Song P., Li B., Tang Z., Hu F., Shi X. (2022). Characterization of chemical composition variations in raw and processed *Codonopsis* Radix by integrating metabolomics and glycomics based on multiple chromatography-mass spectrometry technology. J. Sep. Sci..

[21] Hyun S.H., Bhilare K.D., In G., Park C.K., Kim J.H. (2022). Effects of *Panax ginseng* and ginsenosides on oxidative stress and cardiovascular diseases: Pharmacological and therapeutic roles. J. Ginseng Res..

[22] Hao J. (2012). Quality Evaluation and Influence Factors of Ginseng Product. Master’s Thesis.

[23] Yao F., Li X., Sun J., Cao X.X., Liu M.M., Li Y.H., Liu Y.J. (2021). Thermal transformation of polar into less-polar ginsenosides through demalonylation and deglycosylation in extracts from ginseng pulp. Sci. Rep..

[24] Li M., Chen Y., Xiao W., Cheng S., Liu F., Huang L. (2019). Determination of drying kinetics and quality changes of *Panax quinquefolium* L. dried in hot-blast air. LWT.

[25] Wu Q., Yan Q., Jiang L., Chen C., Huang X., Zhu X., Zhou T., Chen J., Yan J., Wen F. (2023). Metabolomics analysis reveals metabolite changes during freeze-drying and oven-drying of *Angelica dahurica*. Sci. Rep..

[26] Mwadzingeni L., Shimelis H., Tesfay S., Tsilo T. (2016). Screening of bread wheat genotypes for drought tolerance using phenotypic and proline analyses. Front. Plant Sci..

[27] Xia C., Wen P.P., Yuan Y.M., Yu X.F., Chen Y.J., Xu H.Q., Cui G.Y., Wang J. (2021). Effect of roasting temperature on lipid and protein oxidation and amino acid residue side chain modification of beef patties. RSC Adv..

[28] Witte C., Herde M. (2022). Nucleotide metabolism in plants. Plant Physiol..

